# Red Blood Cells Oligosaccharides as Targets for Plasmodium Invasion

**DOI:** 10.3390/biom12111669

**Published:** 2022-11-11

**Authors:** Patrycja Burzyńska, Marlena Jodłowska, Agata Zerka, Jan Czujkowski, Ewa Jaśkiewicz

**Affiliations:** Hirszfeld Institute of Immunology and Experimental Therapy, Polish Academy of Sciences, R. Weigla, 553-114 Wroclaw, Poland

**Keywords:** *Plasmodium* 1, invasion receptors 2, glycans 3

## Abstract

The key element in developing a successful malaria treatment is a good understanding of molecular mechanisms engaged in human host infection. It is assumed that oligosaccharides play a significant role in *Plasmodium* parasites binding to RBCs at different steps of host infection. The formation of a tight junction between EBL merozoite ligands and glycophorin receptors is the crucial interaction in ensuring merozoite entry into RBCs. It was proposed that sialic acid residues of O/N-linked glycans form clusters on a human glycophorins polypeptide chain, which facilitates the binding. Therefore, specific carbohydrate drugs have been suggested as possible malaria treatments. It was shown that the sugar moieties of *N*-acetylneuraminyl-*N*-acetate-lactosamine and 2,3-didehydro-2-deoxy-*N*-acetylneuraminic acid (DANA), which is its structural analog, can inhibit *P. falciparum* EBA-175-GPA interaction. Moreover, heparin-like molecules might be used as antimalarial drugs with some modifications to overcome their anticoagulant properties. Assuming that the principal interactions of *Plasmodium* merozoites and host cells are mediated by carbohydrates or glycan moieties, glycobiology-based approaches may lead to new malaria therapeutic targets.

## 1. Introduction

Malaria remains a major global health problem, being responsible for approximately 500,000 deaths each year globally, mostly among children and pregnant women in sub-Saharan Africa [1]. Long-term sustained control and elimination of malaria requires the development of new drugs and an effective vaccine, which remains an ambitious international goal [2]. The crucial point in developing a successful malaria treatment is a good understanding of molecular mechanisms engaged in human host infection.

Malaria is caused by apicomplexan parasites of the genus *Plasmodium*, which are transmitted via bites of infected female *Anopheles* mosquitoes [3]. The *Plasmodium* species can infect not only humans but apes as well. Humans are infected by *Plasmodium falciparum*; three other species infect chimpanzees (*P*. *reichenowi*, *P*. *gaboni*, and *P*. *billcollinsi*) while the other three infect gorillas (*P*. *praefalciparum*, *P*. *blacklocki*, and *P*. *adleri*) [4,5].

After a bite by an infected mosquito, *Plasmodium* parasites multiply in the liver and then are released in form of merozoites, which infect red blood cells (RBCs) [6]. This asexual erythrocytic phase of the life cycle of parasite produces all of the clinical symptoms of malaria [7,8]. The invasion of RBCs by *Plasmodium* merozoites can be divided into multiple steps in which several protein ligands expressed by merozoites are involved [9,10,11,12]. Initial attachment of the parasite to the RBC is followed by reorientation to bring merozoite’s apical end into close contact with the surface of the erythrocyte. The parasite forms a junction between its apical end and the RBC surface. Junction formation is irreversible and is followed by the entry of merozoite through invagination of the RBC membrane. The process of junction formation is mediated by interaction between parasite ligands and specific RBC receptors. Parasite mediators of invasion have been identified on the merozoite surface [13]. Among them, erythrocyte binding-like (EBL) proteins play a crucial role in the attachment of merozoites to human RBCs by binding to specific receptors on the red cell surface [14]. Four homologous *P*. *falciparum* EBL ligands were identified [15] (Table 1). The major EBA-175 merozoite ligand [16] recognizes glycophorin A (GPA) [17,18], the EBA-140 ligand [19,20] binds to minor erythrocyte glycophorin C (GPC) [21,22,23,24], the EBL-1 ligand prefers glycophorin B, and the receptor preferred by the EBA-181 ligand still remains unknown (Table 1).

Glycophorins are major sialoglycoproteins of human and animal RBCs, with relatively low molecular weight (20–30 kDa), carrying sialylated O-glycans and/or N-glycans [14]. Most GPA and GPB O-glycans are NeuAcα2-3Galβ1-3(NeuAcα2-6)GalNAc—tetrasaccharide chains linked to serine or threonine residues [25,26]. However, monosialylated linear trisaccharide and sialylated or non-substituted GalNAc residues are also present. It is suggested that all human glycophorins contain similar O-glycans. GPA and GPC also contain N-glycosidic chain attached to Asn26 and Asn8 residues, respectively. The structures of both N-glycans have been determined [27,28,29]. They contain complex biantennary chains with a bisecting GlcNAc and terminal sialic acid residues. In addition, GPC N-glycan contains small amounts of terminal fucose [29]. Recent genome-wide association studies have shown that resistance to malaria is connected with human glycophorin *ABE* locus [30], confirming that glycophorins play an important role in erythrocyte invasion by *Plasmodium* merozoites.

In addition, alternations of the O-glycosylation pathway in beta-thalassemic RBCs were identified as an increase in O-GlcNAcylation of serine and threonine residues [31]. This haemoglobinopathy is caused by mutations, which reduce or eliminate beta globin production from the beta globin gene (*HBB*) [32]. Heterozygosity for beta-thalassemic mutations manifests only in a mild anemia, but homozygosity can cause a life threatening condition. The disease is associated with malaria prevalence [33] and was purported as being meaningful in malaria protection. O-GlcNAcylation is a common post-translational modification in *P. falciparum* proteins following malaria infection; thus, a therapeutic blockade of this pathway has been proposed to shorten the life cycle of the *Plasmodium* [34]. This observation might explain the molecular mechanisms of beta-thalassemia protection from malaria infection.

In this review, we present the role of sugars as major mediators of merozoite–ligands interaction and suggest that they may determine *Plasmodium* host specificity.

## 2. Sialic Acids

Sialic acids are terminal carbohydrate moieties on cell glycocalyx. Most of them are linked to glycoproteins, except for human neuronal cells, where sialic acids are linked to sphingolipids (gangliosides) [35]. As terminal, hydrophobic molecules with a negative charge, they are involved in cell stability and interactions with other cells and pathogens or toxins as well [36,37,38]. 

**Figure 1:**
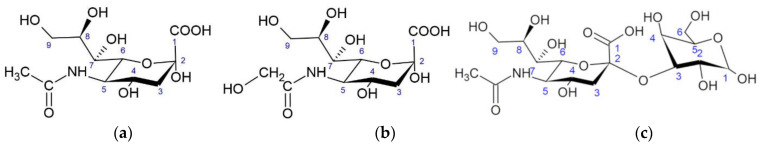
Structure of sugar receptors of EBL ligands: (**a**) *N*-acetylneuraminic acid (Neu5Ac), (**b**) *N*-glycolylneuraminic acid (Neu5Gc), (**c**) α(2 → 3) Sialyllactose.

Sialic acids are derivatives of neuraminic acid (nonulosonic acid) [39]. There are two major forms of sialic acid: *N*-5-acetylneuraminic acid (Neu5Ac) and *N*-5-glycolylneuraminic acid (Neu5Gc) (Figure 1) [35,36,37,38,39,40]. Neu5Gc arises from Neu5Ac by hydroxylation of its acetyl moiety in a reaction catalyzed by cytidine monophosphate-N-acetylneuraminic acid hydroxylase (CMAH, encoded by *CMAH* gene) [41,42]. The *CMAH* gene is nonfunctional in New World monkeys, so European hedgehog, *Mustelidae*, *Procyonidae*, some bats, sperm whale, white-tailed deer, and platypus [42,43,44] do not express Neu5Gc. Humans also belong to this group of species as the *CMAH* gene ceased to encode active protein some 2 million years ago [44,45,46,47,48].

The linkage configurations of sialic acids are α(2,3) or α(2,6) with galactose or *N*-acetylgalactosamine and α(2,8/9) in polysialic acids. These bonds are created by specific sialyltransferases and polysialyltransferases, respectively [49]. The α-glycosidic bond is hydrolyzed by hydrolases, which are named neuraminidases or sialidases [50,51].

**Figure 2:**
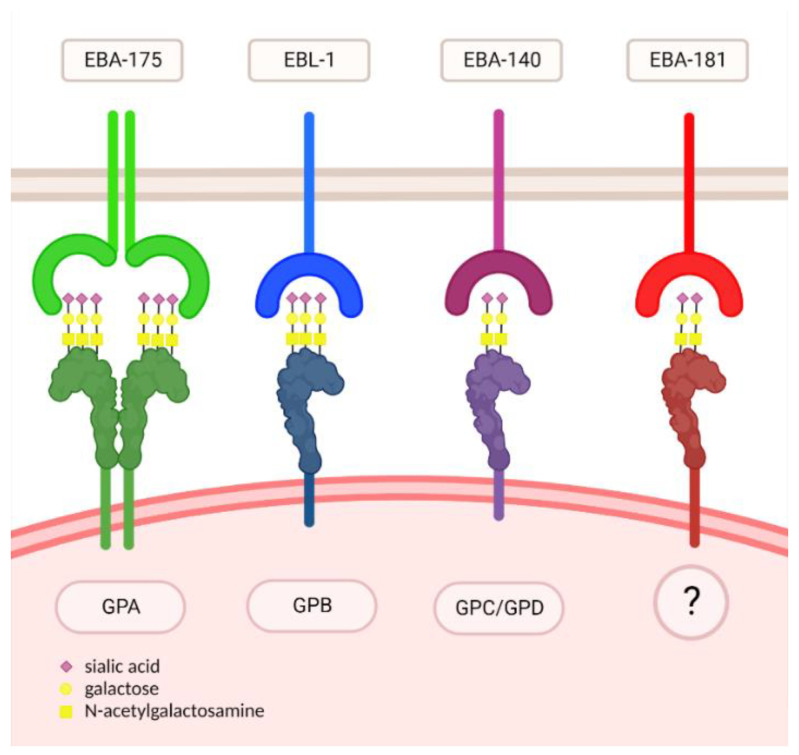
*P. falciparum* EBL merozoite ligands and glycophorin receptors on RBCs.

It was shown previously that EBA merozoite ligands do not bind to RBCs treated with neuraminidase, which cleaves terminal α(2,3/6)-linked sialic acids from oligosaccharide chains [52]. Since the majority of sialic acid residue on O-glycans is linked to glycophorins, it may be argued that sialic acids play a crucial role in the *Plasmodium* merozoites binding to RBCs [14]. The crystal structure of the erythrocyte-binding domain (RII) of *P*. *falciparum* EBA-175 ligand was resolved, giving insight into the molecular mechanisms underlying the GPA binding [53]. The dimer of RII was also co-crystallized with a glycan (α-(2,3)sialyllactose, Figure 1) that may be considered as an analogue of Neu5Acα(2,3)-Gal (the O-glycan on GPA). The RII dimer interface contains six glycan-binding sites, so it seems that RII binding depends on its dimerization. A mutational analysis of RII suggested that all six glycan-binding sites are necessary for RII binding. All these data helped to create a binding model of EBA-175 to GPA in which the binding induces dimerization of RII by assembling around the dimeric GPA extracellular domain. In summary, it is now generally assumed that α(2,3)Neu5Ac residues of O-linked glycosaccharides form conformation-dependent clusters on GPA polypeptide chains, which facilitates binding [17,18] (Figure 2).

EBL-1 is another homologous ligand that failed to bind to RBCs treated with neuraminidase, as was chymotrypsin, which cleaves GPB, which was shown as the RBCs receptor for EBL-1 [54]. Therefore, it was suggested that GPB with sialylated O-glycans, such as GPA for EBA-175, may serve as a receptor for EBL-1 [14] (Figure 2). 

Studies on the third *P*. *falciparum* EBA-140 homologous ligand [19,20] showed that it also does not bind to the neuraminidase-treated human RBCs. The crystal structure of its RII bound to sialyllactose [55,56], confirming the interaction of this ligand with sialic acid. Two receptor glycan-binding sites, located in the RII homologous domains F1 and F2 in a structurally similar position, were revealed. Thus, the EBA-140 ligand can bind only to two GPC glycans (Figure 2), whereas the PfEBA-175 RII dimer interacts with six O-linked glycans of GPA. Moreover, the crucial role of GPC sialylated N-glycan for GPC-EBA-140 interaction was shown [29,57]. Thus, the essential role of sialylated O- and N-oligosaccharide chains on GPC in EBA-140 binding was generally established [58]. The ape homologue of the *P*. *falciparum* EBA-140 has been identified in chimpanzee *P*. *reichenowi*. We have shown that the recombinant ape EBA-140 RII specifically recognizes sialylated oligosaccharides on chimpanzee RBCs [59]. The glycophorin D (a truncated form of GPC) was identified as receptor for *P*. *reichenowi* EBA-140 ligand [60] (Figure 2).

In addition to sugar moieties, the GPA, GPB, GPC, and GPD polypeptide chains also play a significant role in the recognition and binding of the EBA-175, EBL-1, EBA-140 ligands, respectively [14,38] (Figure 2).

The specificity of the EBA-181 erythrocyte receptor is still unknown [9,56]. It was shown that neuraminidase and chymotrypsin treatment causes a decrease in the EBA-181 binding to RBC, but trypsin treatment did not influence it [61]. So, the given binding sensitivity profile pointed at GPB. However, the unchanged binding of the EBA-181 ligand to S-s-U-RBCs, that lack GPB, ruled out this possibility. However, the mysterious sialylated glycoprotein receptor still has to be considered (Figure 2). Our studies confirmed sugar specificity of this ligand, similar to other EBA protein family members, since EBA-181 binds to Neu5Ac and Neu5Ac(α2,3)-Gal in the surface plasmon resonance (SPR) method (unpublished results). However, the exact RBC membrane receptor carrying such sugars remains to be uncovered.

The ape homologues of the EBA P. falciparum ligands, including chimpanzee *P*. *reichenowi*, have been identified [60,62]. It was shown that the receptors for *P*. *reichenowi* EBA-175 and EBA-140 ligands are N-glycolylneuraminic acid (Neu5Gc), while *P. falciparum* EBA-175 and EBA-140 bound to Neu5Ac [63]. Thus, it may be argued that the species specificity of EBA-175 and EBA-140 depends on the type of sialic acid molecules [47,64]. However, further studies on the *P. falciparum* EBA-175 ligand revealed the strong binding of EBA-175 to Neu5Gc monosaccharide as well [65,66,67]. Thus, there is a general agreement that EBA-175 can utilize both Neu5Ac and Neu5Gc.

EBA-165, another homologous merozoite protein, is present in all ape-infective species, but due to a 5′ frameshift in genes, which truncates the transcript before the RII binding domain, is not expressed in human-infecting *P*. *falciparum* [67]. It was shown that *P*. *reichenowi* EBA-165 bound only to glycans containing Neu5Gc [67].

In conclusion, there is a clear preference of the binding to Neu5Gc in all studied ape species, while human *P*. *falciparum* shows the specificity towards both Neu5Ac and Neu5Gc. It suggests that *P. falciparum* has gained the ability to bind Neu5Ac without losing the binding specificity for Neu5Gc. 

It is generally assumed that individuals with sickle cell disease, which is caused by a point mutation in the β-globin gene and manifests by chronic anemia, hemolysis, and inflammation, are more resistant to malaria than non-sicklers [68]. It has already been observed that RBCs sialic acids content is significantly lower in sickle-cell individuals than in healthy ones [69,70]. However, recently it was shown that sickle cell RBCs are characterized by an increasing level of α(2,6)-linked sialic acids but a decreasing level of α(2,3)-linked sialic acids [71], which were identified as receptors for EBA merozoite ligands, as was mentioned previously [17,65].

Thus, it may argued that *Plasmodium* malaria merozoites exploit the diversity of sialic acids on the surface of RBC to invade their hosts.

## 3. Antigens of Human ABO Blood Group System

**Figure 3:**
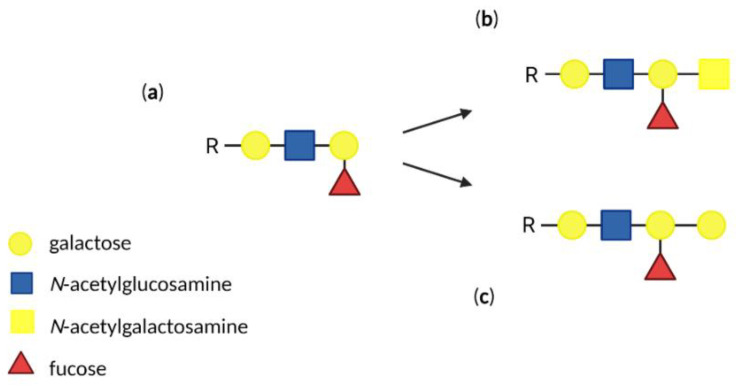
Structures of ABH blood group antigens: (**a**) H antigen, (**b**) A antigen, (**c**) B antigen. Created with BioRender.com.

ABO blood group antigens are expressed not only on the RBCs but also on epithelial and endothelial cells. ABO blood group phenotype is determined by a presence on the RBCs surface of the A, B, H blood group antigens, respectively. These antigens are trisaccharides, which consist of N-acetylgalactosamine, galactose, and fucose residues (Figure 3). It has been shown that ABO blood group may influence P. falciparum erythrocyte invasion [72]. A risk of severe malaria and death is significantly higher in individuals with A, B, and AB blood groups, in comparison with those in the O blood group [73]. Generally, it is now approved that ABH group antigens participate in two cytoadherence phenomena called “rosetting” and “sequestration” [74].

Rosetting is the adhesion of infected RBCs to other uninfected peripheral RBCs [75]. It was shown that in infected individuals in the A blood group, rosettes are larger, more stable, and occur more frequently than rosettes formed in the O blood group individuals [76,77,78]. Moreover, rosettes formed with all non-O blood group RBCs show reduced accessibility for anti-PfEMP1 antibodies [79]. Thus, it is assumed that rosetting may play a role in the avoiding of immune surveillance and in keeping close ready to infect RBCs [80]. A difference in the rosetting of subgroups A_1_ and A_2_ in RBCs was also found [79,81]. The RBCs of the A_1_ subgroup express approximately four to five times more A antigens than those of the A_2_ subgroup [82]. Therefore, rosettes made with RBCs of A_1_ phenotype were larger and less susceptible to anti-PfEMP1 antibodies and the damaging effect of heparin than A_2_-phenotype RBC rosettes [79]. Moreover, the enzymatic removal of terminal sugars, D-GalNAc and D-Gal, in A and B antigens, respectively, results in smaller, weaker rosettes, similar to those with group O erythrocytes [83,84]. However, rosettes still form with O group RBCs, expressing only H antigens and even with RBCs from individuals with a Bombay phenotype in which A, B, and H antigens are absent [75,83].

Sequestration is the adhering of infected RBCs to endothelial cells, which occurs when young parasites reach the trophozoite stage [74]. It reduces the amount of blood flow in vessels, causing hypoperfusion and inflammation in host organs and makes infected RBCs more resistant to the host immune system [80]. It may also cause parasite accumulation in the placenta, which results in clinical consequences [85]. It was proposed that P. falciparum erythrocyte membrane protein-1 (PfEMP-1), the main adhesin belonging to the VAR protein family present on infected RBCs [81], is responsible for the rosetting and sequestration phenomena. The direct binding of the VarO (PfEMP1 adhesion ligand) to the blood group A and B antigens was shown in SPR [81].

The genes belonging to two multigene families encode RIFIN and STEVOR proteins that are expressed on the surface of the infected RBCs, which implies that they may serve as potential cytoadherence-mediating virulence factors [86,87]. RIFIN can form rosettes by binding to RBCs of blood group A and such rosettes are larger than those formed with RBCs of blood group O [88]. STEVOR mediates rosetting independently of PfEMP1 and RIFIN through interaction with GPC on the surface of the uninfected RBCs [86]. Other receptors, such as as CD31, CD36, immunoglobulin M, complement receptor 1 (CR1), and glycosaminoglycans (GAGs), such as heparan sulfate (HS), are assumed to have a role in cytoadherence apart from the ABO(H) blood group system antigens [75,89].

## 4. Glycosaminoglycans (GAGs)

**Figure 4:**
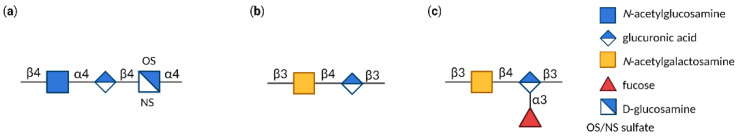
Structures of glycosaminoglycans (GAGs): (**a**)/heparan sulfate (HS), (**b**) chondroitin sulfate (CS), (**c**) fucosylated chondroitin sulfate (FucCS). Created with BioRender.com.

Glycosaminoglycans are components of mammalian cells extracellular matrix [90]. Heparin and heparan sulfate (HS) are linear GAGs, consisting of repeating disaccharide units D-glucuronic acid β (1–4) and N-Acetyl D-glucosamine (Figure 4). Heparin and HS molecules are located on the surface of RBCs [91,92].

Full-length *P*. *falciparum* merozoite surface protein 1 (MSP-1), the most abundant merozoite surface protein, is responsible for the initial contact with RBCs [93]. MSP-1 and its proteolytic fragments MSP-142 and MSP-133, which are shed from the merozoite surface during invasion, can bind to heparin-like molecules on RBCs. It was shown that heparin can inhibit the schizont rupture and merozoite invasion by 80% [94].

*P*. *falciparum* EBA-140 ligand contains several potential GAG-binding motifs and blockages of these motifs by soluble heparin or HS induce an inhibition of this ligand binding and the merozoite invasion [95]. HS was shown as an RBC receptor for EBA-140 ligand together with GPC. It was suggested that the binding of EBA-140 to RBCs is mediated primarily by GPC sialic acid residues and partially through HS. Thus, the small amount of HS supports the sialic acid-dependent GPC binding of EBA-140 and facilitates merozoite invasion. 

Moreover, HS on hepatocytes was suggested to bind to *P*. *falciparum* circumsporozoite protein (CSP), mediating the transmission of parasite to liver cells [96,97].

Additionally, HS is a receptor for *P*. *falciparum* erythrocyte membrane protein 1 (PfEMP1), expressed on parasite-infected RBCs, mediating the binding of infected RBC to the vascular endothelial cells or other RBCs [98,99].

Although the role of HS as an RBC rosetting receptor requires confirmation, it was shown that heparin and other GAGs, due to their rosette-disrupting effects have clear potential as adjunctive therapies for severe malaria [75].

The first approach in using heparin in malaria therapy was attempted half a century ago [100], but the main problem was bleeding, due to its anticoagulant properties. Thus, depolymerized heparin with low or lacking anticoagulant effects has been proposed as a treatment to reduce rosetting and sequestration in malaria [101]. Recently, chemically modified heparin-like molecules (HLMs) with antimalarial activity but low anticoagulating effect, due to hypersulfation, have been developed [102]. Moreover, semi-synthetic, heavily sulfated, non-GAG HLMs as glycogen type 2 sulfate and phenoxyacetylcellulose sulfate were also successfully studied [103].

Chondroitin sulfate (CS) is a linear GAG polysaccharide, consisting of β(1-3)*N*-Acetyl-D-galactosamineβ(1-4)glucuronic acid units (Figure 4). *Plasmodium* merozoites binding to chondroitin sulfate A (CSA) in the placenta may lead to the accumulation of parasites causing severe symptoms called placental malaria [104]. This is associated with increased risks of adverse obstetric outcomes, including maternal anemia, preterm delivery, fetal growth restriction, low birth weight, and maternal and neonatal mortality [105]. The interaction occurs trough the VAR2CSA protein from VAR-family of proteins expressed on the surface of infected RBCs [106,107]. 

Moreover, cytoadhesion and invasion inhibitory effects were also reported for natural HLMs, fucosylated chondroitin sulphate (FucCS), isolated from sea cucumber [108]. The presence of sulfated fucose branches was crucial for the biological effects of FucCS. Other HLMs with less anticoagulant activity than heparin were obtained from red algae and marine sponges [109]. These findings demonstrate that synthetic and natural HLMs might be considered for the treatment of malaria complications during pregnancy.

## 5. Conclusions

In conclusion, it is now generally assumed that human and ape *Plasmodium* merozoites use host oligosaccharides in the invasion of RBCs. It was shown that sugars are the significant mediators of the merozoite ligands binding to RBC receptors at different steps of host infection. The key interaction ensuring merozoite entry into RBCs is the formation of the tight junction between the EBA ligands and glycophorin receptors. It was suggested that sialic acid residues of O/N-linked glycosaccharides form a conformation-dependent cluster on human glycophorins polypeptide chains, which facilitates the binding. Therefore, specific approaches with carbohydrate drugs have been proposed as possible malaria treatments [110].

It was shown that the sugar moieties of *N*-acetylneuraminyl-*N*-acetate-lactosamine and 2,3-didehydro-2-deoxy-*N*-acetylneuraminic acid (DANA), which is its structural analog, can inhibit *P*. *falciparum* EBA-175-GPA interaction in 81% and 84% at 100 uM, respectively [111]. Moderate inhibition was also observed for monomers or oligomers of *N*-acetylneuraminic acid (from 25 to 68% at 100 uM), in comparison with full GPA (88%). Moreover, it was shown that DANA is able to inhibit *P*. *falciparum* RBCs invasion (50–60% at 5000 µM). Thus, such compounds might be used as part of drug cocktails to reduce disease severity.

Considering the emerging problem of the antimalarial drug resistance, including quinolines, chloroquine, antifolate, and most recently artemisinin [112,113], glycans seem to be the rational choice for complementary malaria treatments. In particular, synthetic and natural HLMs seem to be promising antimalarial drugs and might be used due to their reduced anticoagulant properties [102]. For instance, HLM Sevuparin, an acidic, negatively charged, anti-adhesive polysaccharide drug, manufactured from heparin with an eliminated antithrombin-binding site, affects both merozoite invasion and sequestration of infected RBCs. The drug was shown in phase I/II human clinical trials to be safe and well tolerated [114]. Sevuparin acts as decoy receptor blocking invasion and rapidly, transiently de-sequestering infected RBCs, which are beneficial in patients with *P. falciparum* severe and complicated malaria.

Another approach was using a vaccine for the inhibition of *Plasmodium* binding to placenta [115]. The vaccine candidate PAMVAC was based on the recombinant fragment of VAR2CSA, the *P. falciparum* protein responsible for binding to the placenta via CSA. PAMVAC vaccine formulated with Alhydrogel or GLA-based adjuvants was safe, well tolerated, and induced functionally active antibodies in healthy malaria-naive adults. Next, PAMVAC will be assessed in women before first pregnancies in an endemic area.

In summary, a fresh approach, based on glycobiology, may lead to new malaria therapeutic targets, since the principal interactions of *Plasmodium* merozoites and host cells are mediated by carbohydrates or glycan moieties [110,116].

## Figures and Tables

**Table 1:** *P. falciparum* EBL merozoite ligands and glycophorin RBC receptors.

Ligand	Receptor	Oligosaccharide
EBA-175	GPA	Neu5Ac(α2,3)-Gal-
EBL-1	GPB	Neu5Ac(α2,3)-Gal-
EBA-140	GPC/GPD	Neu5Gc(α2,3)-Gal-
EBA-181	?	Neu5Gc(α2,3)-Gal-
